# A systematic review of the impact of routine collection of patient reported outcome measures on patients, providers and health organisations in an oncologic setting

**DOI:** 10.1186/1472-6963-13-211

**Published:** 2013-06-11

**Authors:** Jack Chen, Lixin Ou, Stephanie J Hollis

**Affiliations:** 1 The Simpson Centre for Health Services Research, South Western Sydney Clinical School, University of New South Wales, Liverpool 2170NSW, Australia; 2 Australian Institute of Health Innovation, Level 1, AGSM Building, University of New South Wales, Randwick 2052, Australia

**Keywords:** Cancer, Quality of Life, Patient Reported Outcomes, Health Services Research

## Abstract

**Background:**

Despite growing interest and urges by leading experts for the routine collection of patient reported outcome (PRO) measures in all general care patients, and in particular cancer patients, there has not been an updated comprehensive review of the evidence regarding the impact of adopting such a strategy on patients, service providers and organisations in an oncologic setting.

**Methods:**

Based on a critical analysis of the three most recent systematic reviews, the current systematic review developed a six-method strategy in searching and reviewing the most relevant quantitative studies between January 2000 and October 2011 using a set of pre-determined inclusion criteria and theory-based outcome indicators. The Grading of Recommendations, Assessment, Development, and Evaluation (GRADE) system was used to rate the quality and importance of the identified publications, and the synthesis of the evidence was conducted.

**Results:**

The 27 identified studies showed strong evidence that the well-implemented PROs improved patient-provider communication and patient satisfaction. There was also growing evidence that it improved the monitoring of treatment response and the detection of unrecognised problems. However, there was a weak or non-existent evidence-base regarding the impact on changes to patient management and improved health outcomes, changes to patient health behaviour, the effectiveness of quality improvement of organisations, and on transparency, accountability, public reporting activities, and performance of the health care system.

**Conclusions:**

Despite the existence of significant gaps in the evidence-base, there is growing evidence in support of routine PRO collection in enabling better and patient-centred care in cancer settings.

## Background

Patient reported outcome (PRO) measures include health status assessments and measures for health-related quality-of-life (HRQOL), symptom reporting, satisfaction with care, treatment satisfaction, economic impact, and specific dimensions of patient experience such as depression and anxiety [1]. The USA Food and Drug Agency (FDA) adopts a much broader definition [2] as *“A PRO is any report coming directly from patients about a health condition and its treatment”*, meaning that PROs capture patients’ perspectives about how illness or new therapies impact on their general well-being. There is a growing interest from clinicians, researchers, industry and policy-makers in routinely collecting PROs to facilitate timely, patient-centred and evidence-based care. For example, the National Health Service (NHS) of the UK has been implementing a world-leading initiative for the routine collection of PROs that firstly included a few selected elective surgeries (e.g. unilateral hip replacements, unilateral knee replacements, groin hernia surgery or varicose vein surgery) [3] but are soon expanding to many other conditions such as mastectomy and breast cancer, among others. In the USA, the Patient-Reported Outcomes Measurement Information System (PROMIS), a National Institutes of Health funded initiative starting in 2004, is providing a publicly available web-based resource that can be used to measure key health symptoms and HRQOL [4]. The traditional paper-based PROs instruments are limited by its lack of flexibility, language and literacy requirement, [5,6] possible inappropriateness towards minority groups, [7,8] lack of timeliness (in generating instantaneous clinical meaningful interpretations) [9] and inability to adopt state-of-the-art measurement science such as Item Response Theory (IRT) and Computer Adapted Test (CAT) technique [10]. To overcome the difficulty of integrating the administration and analysis of PRO instruments into clinical practice, researchers are developing and validating alternatives to traditional paper-based instruments such as office-based touch-screen computers, [11-13] telephone-based interactive voice-response (IVR) systems, [14-16] hand-held computers, [17,18] mobile phones, [19-21] and more recently, the Internet [22-24]. Some rationales [25-28] put forward for measuring PROs in a cancer setting include, but not limited to: 1) better communication and shared decision making by patients and providers; 2) assessing the health status of patients entering therapy and identifying treatable problems; 3) determining the degree and sources of the patient’s decreased ability to function; 4) distinguishing between types of problems, including physical, emotional, and social; 5) detecting adverse effects of therapy; 6) monitoring the effects of disease progression and response to therapy; 7) informing decisions about changing treatment plans, and 8) predicting the course of disease and outcomes of care.

However, despite growing interest and urges by the leading experts for applying routinely collected PROs for all cancer patients, there has not been an updated comprehensive review of the evidence regarding the impact of adopting such a strategy on patients, services providers and organisations. The most recent review focused only on clinical trial design [26] studies of cancer patients, and only assessed a limited number of outcomes. The current project aims to provide the much needed comprehensive review update, including all relevant quantitative studies investigating the effectiveness of routine PRO collection in cancer patients. The review research questions were:

1. What are the impacts of composite measures of PROs collected on cancer patients during treatment with regards to:

a) Provider behaviour for improving care delivered;

b) Organisational changes within health care settings for improving processes and models of care (e.g. targeting and tailoring care);

c) Improving clinical outcomes for patients; and

d) Improving patient experience of care (e.g. self-care).

2. What mechanisms are involved in the link between PROs and the impacts identified in 1(a)?

3. What factors moderate the extent of the impacts identified in 1(a)?

## Methods

### Existing systematic reviews and rationale for the current review

In order to develop an efficient search and review strategy, over 200 existing reviews on the same or similar topics were firstly systematically examined (identified in a broad search covering PROs and quality of life measures between January 2000 and October 2011). Three reviews [26-28] were identified as the baseline reviews for this project and their review strategies were carefully examined in aspects such as the aim and scope, time span, search strategy and search terms used, articles included in each review, and conclusions drawn. A table summarising the three systematic reviews is presented in Table 1.

**Table 1:** A comparison of three baseline reviews

**First author, year**	**Aim and review scope**	**Time span and the search strategy**	**Search Terms**	**Articles included in the review**	**Major conclusions**
Luckett et al. 2009 [26]	To identify future strategies for (1) interventions to impact patient outcomes; and (2) trials to identify treatment effects.	MEDLINE and PsycINFO were systematically searched to identify reports of relevant randomised controlled trials. The time span was between 2006 and 1 August 2008. Four cancer trials were cited in a previous review (Valderas et al. 2008)[27].	1. Examined the citations of the four trials 2. Adopted the strategy used by Valderas et al. [27] and Espallargues et al. [29] which involved searching for the terms ‘health status’, ‘functional status’ or ‘quality of life’ and ‘clinical practice’, ‘clinical setting’, ‘practice setting’, ‘medical practice’ or ‘medical consultation’ anywhere in the title, abstract or keywords. Results were limited by publication date (2006–2008) and the MeSH or keyword neoplasm.	6 RCTs	Future interventions should motivate and equip health professionals to use PROs data in managing patients, training patients in self-efficacy, using more specific PROs in clinics, improving the interpretability of feedback for both medical staff and patients, and monitoring the use of PROs to intervene when problems arise. Future trials should use a cluster randomised design to control for contamination and enable systems-based interventions.
Valderas et al. 2008 [27]	To summarize the best evidence regarding the impact of providing patient reported outcomes (PRO) information to health care professionals in daily clinical practice.	Systematic review of randomised clinical trials (Medline, Cochrane Library); reference lists of previous systematic reviews; and requests to authors and experts in the field. Time span: Articles published between 1978 and 2007.	No exact search terms provided but indicated available from the author upon request.	34 articles corresponding to 28 original studies; only 2 (not 4) as mentioned in the above review, are in an oncologic setting.	Methodological concerns limit the strength of inference regarding the impact of providing PROs information to clinicians. Results suggest great heterogeneity of impact; contexts and interventions that will yield important benefits remain to be clearly defined.
Marshall et al. 2006 [28]	To synthesize the evidence for using publically reported performance data to improve quality. Only articles that provided empirical evidence on the impact of public reporting on outcomes (effectiveness, patient safety, and patient-centeredness) and unintended consequences, as well as selection and quality improvement activity were included.	Webspirs Medline was searched for the years from January 1976 to November 2004. Reference lists of included studies and appropriate reviews (Greenhalgh & Meadows 1999[30]; Espallargues et al. 2000 [29]; Gilbody et al. 2003 [31]) were also searched for relevant articles. Finally, PubMed’s ‘related articles’ feature was used with several background and included articles (Drury *et al.* 2000 [32]; Velikova et al. 2004 [13]) to identify publications with a high proportion of similar text in the title and abstract.	Terms used in relation to patient-reported outcome measures (for example, ‘self report* near2 measure*’) joined with an ‘and’ command to terms related to routine practice outcomes (for example, ‘improve* near detect*’) or patient involvement in the health care process (such as ‘patient* near provider* near interaction*’).	40 articles included in the review including 5 publications from an oncologic setting.	The pattern of results suggests a general lack of clarity in the field, especially regarding appropriate goals for PROs and the mechanisms by which they might achieve them. To fully evaluate their role in routine practice, studies need to use PROs that capture issues of importance to patients and to measure impacts relating to the patient–provider relationship and patient contributions to their well-being. Until studies evaluate PROs as a means to facilitate patient-centred care, their full potential in clinical practice will remain unknown.

### Review search strategy

Analysing the results of above three systematic reviews demonstrates the importance of search strategies in determining what literature will be included in the study, which in turn, may influence what conclusions will be derived. Valderas et al.’s (2008) [27] review excluded three out of the five clinical trials on cancer patients that were included in Marshall et al.’s (2006) [28] review. Lucket et al.’s (2009) review [26] excluded one article (Taenzer et al. (2000), [33] a before-after study) from Marshall et al.’s review [27]. A mixed methodology search was developed in order to maximise the identification of recent literature in a short period of time. The search was conducted in six different ways as follows:

1. A text-based search strategy was developed based on previous reviews. To elicit previous reviews, a search was conducted for the text terms ‘patient reported outcome*’, ‘self-reported’, ‘self-assessed’ anywhere in title, abstract and key words, combined with ‘quality of life’, ‘symptom’, ‘functional status’, ‘health status’, ‘patient satisfaction’, ‘unmet need*’ anywhere in title, abstract and key words. For original articles, a search was conducted using the same strategy as above but restricted to those with ‘neoplasm’ or ‘cancer’ in the key words. The search results were restricted to between January 2000 and October 2011 (full search strategy is listed in Additional file 1: Appendix 1).

2. All reviews were evaluated (over 200 in total on various topics but not limited to only cancer patients) with three baseline reviews used as the starting point for our top-down and bottom-up search strategy. We chose the three baseline reviews because that: 1) they are all systematic reviews that could be helpful in forming the structure or strategy of the current review (but not necessarily restricted to cancer patients); and 2) they were published after 2005.

3. All articles were examined if they cited the 7 key randomised controlled trials [33-39] listed in the above reviews (bottom-up approach). References were also sought from the most recently published trials, editorials, and commentaries (a top-down approach). The powerful citation tracking feature of Scopus™ made this strategy feasible.

4. Simplified text terms (i.e. patient reported outcome, PRO, PROM, Quality of life, QOL) were used to conduct a web search for identifying grey literature.

5. Leading researchers and experts in the field (elicited through the advice of Cancer Institute NSW (CINSW), editorials, review articles and most cited articles) were purposefully searched in order to analyse the references and citations in their publications.

6. Some key cancer centres’ websites were also searched in order to get more detailed information.

The search was limited to the Scopus™ database as it is the largest abstract and citation database of peer-reviewed literature and quality web sources including 100% coverage of Medline titles and EBASE. It also tracks, analyses and visualises publication results, which is well suited to our top-down and bottom-up search strategy.

### Aim, study selection and endpoints of the review

**Figure 1:**
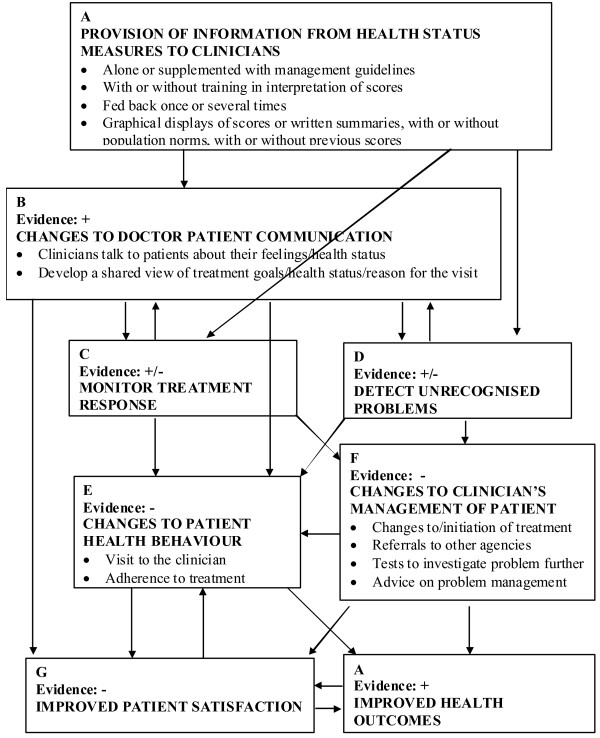
**A hypothetical framework to understand the impact of routinely collected PROs on patient health outcomes (adopted from Greenhalgh et al. (2005) **[25]**with permission).**

In this review, the aim was to synthesize the evidence in relation to the impact of routinely collected PROs on patients, providers, and health organisations. The frameworks proposed by Greenhalgh and colleagues [25] and by Abernethy and colleagues [40] were adopted to guide our evaluation of the existing literature. Greenhalgh et al. [25] proposed a framework (Figure 1) that depicts mechanisms between the routine collection of PROs and changes in patient outcomes. The authors suggest that the multilayer mediators (i.e. changes to doctor-patient communication, monitoring treatment responses, detecting unrecognised problems, changes to patient health behaviour, changes to clinicians’ management plans, and improved patient satisfaction) have complex relationships among them. The studies that revealed these complex relationships may assist in understanding whether and how the underlying mechanisms of routinely collected PROs work to improve the intended outcomes.

**Figure 2:**
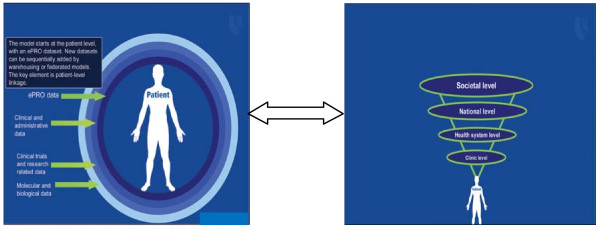
**(a) A data linkage framework (b): A learning health care system.** Note: Figures 2: adopted from Aberthnethy et al. (2010) [40] with permission.

Recently, Abernethy and colleagues [40] have argued that the routine collection of PROs has the capacity to impact not only at the patient-level, but by addressing the logistics of data linkage, and could ensure that the system will grow to accommodate other clinical- and health system-level issues; for example, evaluating comparative effectiveness of treatments, monitoring quality of care, and translating basic science findings into clinical practice (Figure 2). The integration of data systems will fuel rapid learning cancer care at the national and societal levels (see Figure 2a and b), making many types of research and system learning possible across institutions and health sectors. The benefits and implications of such a rapid learning health care system may include, but is not limited to, strong and effective quality improvement (QI), increased transparency, accountability, public reporting, better health system performance (monitoring, planning, financing, evaluating, responding) and better quality of care.

Combining both frameworks, a list of outcome indicators was developed (Table 2) against which each eligible study was assessed. To include not only the doctors’ experience with patients after collecting PROs, but also the experience of other health services providers (i.e. nurses, allied health workers), the term ‘Patient-provider communication’ was used instead of ‘doctor patient communication’ as proposed by Greenhalgh et al. [25]. In order to answer review questions 2 & 3 for the studies included, all possible explicit mediation effects were reviewed through examining if a path-analysis or a mediation-analysis by multiple, staged regression approach was presented in the paper. To examine potential moderating effect, each study was examined to determine if it explicitly tested the interaction effect/moderating effect, or inexplicitly conducted subgroup analysis. Significant possible mediating or moderating effect results were indicated as part of review endpoints in Table 3. Inferences made and the discussion were based on these results.

**Table 2:** Outcome indicators assessed for each eligible study included in the review

**Number**	**Outcomes**
1	Patient-provider communication
2	Monitor treatment response
3	Detect unrecognised problems
4	Changes to patient health behaviour
5	Changes to patient management
6	Improved patient satisfaction
7	Improved health outcomes
8	Strong & effective quality improvement
9	Increased transparency, accountability, public reporting
10	Better system performance (monitoring, planning, financing; evaluating, responding)
11	Mediating variables on the effect (both at individual and organisation level)
12	Possible subgroup effects

Note: Both 11 & 12 are combined in the summarising tables as few studies have explored such issues.

**Table 3:** The characteristics of design and study quality

**Reference**	**Country / Jurisdiction**	**Design**	**Sample / Population**	**Outcome measures**	**PROS used**	**Intervention / Number of times feedback**	**Members of medical team given feedback**	**Management plan offered to team**	**Training to staff**	**Domain 1**	**Domain 2**	**Global Rating**
Trowbridge et al. (1997) [39]	USA (Central Indiana Community Cancer Centres, Indianapolis)	RCT: Intervention / Control	320 cancer outpatients, 13 oncologists and 23 clinics	Pain Management Index(ref); pain medication level (0–3) minus pain level: Patient assessment of pain, pain regiments and relief received Patterns of analgesic prescription	Estimates of average and worst pain over the previous 7 days, satisfaction with current pain regimens and degree of relief received	One	Doctors only (12)	No	No	**	***	√√
Tazenzer et al. (2000) [33]	Canada (Tom Baker Cancer Centre , Calgary, Alberta)	Before-after trial: usual care group /Intervention group with before as control	53 lung cancer patients attending an outpatient lung cancer clinic	EORTIC QLQ-C30 11-item Patient Satisfaction Questionnaire (PDIS) ( adapted through Falvo and Smith,1983) Exit Interview (patient’s perception if QL issues had been addressed during the visit) Medical Record Audit on patients’ care plan	EORTC QLQ-C30 (on a PC)	Once	Doctors and nurses	No	Ground round introduction and training	*	***	√√
McLachlan et al. (2001) [38]	Australia (Peter MacCallum Cancer Centre, Melbourne)	RCT: Intervention /control (ratio: 2:1)	450 cancer patients attending ambulatory clinics	Patient HRQoL (EORTC QLQ-C30) 32-item Patient needs (Cancer Needs Questionnaire Short Form [CNQ] Patient distress (Beck Depression Inventory (BDI) Patient satisfaction (in 6-month) Services provided for those identified as required by coordination nurse	EORTC QLQ-C30, CNQ, BDI (through a touch-screen PC)	One	Doctor and coordination nurse (numbers not reporter)	Individualised plan developed by coordination nurse in accordance with generic psychosocial guidelines	No	**	***	√√
Detmar et al. (2002) [37]	Netherland (Netherlands Cancer Institute, Amsterdam)	RCT: (Cross-over design) Intervention/control	214 palliative cancer patients in a outpatient clinic of a cancer hospital	Patient-doctor communication Doctor’s awareness of patient HRQoL Patient management Patients’/doctors’ satisfaction Patient HRQoL (SF-36) Patients’/doctors’ evaluation of intervention	EORTC QLQ-30	Three	Doctors (n=10)	No	Doctors given 30-mins training and patient mailed a leaflet	***	****	√√√
Mooney et al. (2002) [16]	USA (University of Utah, Salt Lake City, Utah)	A pilot Prospective study over a month period with daily measures	27 patients receiving cancer chemotherapy at a cancer centre outpatient clinic	Telephone-Linked Care system for Chemotherapy (TLC-Chemo Alert) Seven symptoms (nausea and vomiting, fatigue, trouble sleeping, sore mouth, fever, feeling blue, feeling anxious) Exit interview	TLC-Chemo Alert	Patients asked to report daily during the cycle and the alerts were sent to providers	Doctors (n=2)	Yes	Patients trained (10 minutes TLC orientation)	**	**	√
Velikova et al. (2004) [36]	UK (Cancer Research UK Clinical Centre – Leeds)	RCT: Intervention /control-attention/control in a ratio of 2:1:1	286 cancer outpatients attending a large cancer centre of a teaching hospital	Patient HRQoL (FACT-G) Discussion of HRQoL issues in consultation Medical actions (decisions on cancer treatment, symptomatic/supportive treatment, investigations and referrals) Non-medical actions(advice on lifestyle, copying and reassurance) Physician checklist assessing the clinical usefulness of PROM data	EORTC QOQ-C30 Hospital Anxiety and Depression Scale (HADS)	Regular clinic visit over an average of 6 months	Doctor (n=28)	No	One to one training and manual provided	***	****	√√√
Basch et al. (2005) [41]	USA (Memorial Sloan-Kettering Cancer Center, New York)	Prospective pilot study of patient online self-reporting of toxicity symptoms	80 patients diagnosed with a gynaecologic malignancy starting a new chemotherapy regimen	Pattern of use of a Self-reported online Symptom Track and Reporting (STAR) system Patient impression of such system based on an exit questionnaire survey Clinician feedback (through survey and team debriefing)	Symptom Track and Reporting (STAR) based on NCI CATAE system	Any clinic visits during 8-wk study period (mean=3, range 1–6) , also possible log in at home during the period	Doctors and study team (n=unreported)	Yes	Training provide to patients but unreported to staff	**	**	√
Boyes et al. (2006) [35]	Australia (Centre for Health Research & Psycho-oncology, University of Newcastle)	Pilot controlled trial: Intervention /control	80 cancer outpatients attending one cancer centre	Patient symptoms Patient anxiety/depression(HADS) Patient needs (Supportive Care Needs Survey[SCNS] Acceptability of intervention to patient and doctors	Symptoms, HADS SCNS	1^st^ consultation – 100% patients: 2^nd^: 83%; 3^rd^: 71%; 4^th^: 60%	Doctors (n=4)	List of patients needs accompanied by suggestions for appropriate referral	None	**	***	√√
Hoeskstra et al. (2006) [42]	Netherlands (Academic Medical Centre, University of Amsterdam)	RCT: Intervention group with symptom monitoring / control	146 palliative cancer patients recruited through two hospitals and local GPs	10 symptoms from the Symptom Monitor Severity of the reported symptom (0–10 score)	Symptom Monitor Extensive Questionnaire	Weekly self-assessed Symptom Monitor at home; Extensive questionnaire every 2-month	GPs (98 times) and medical specialists(96 times)	No	No	**	***	√√
Korniblith et al. (2006) [43]	USA (Dana-Farber Cancer Institute, Boston)	RCT : Telephone Monitoring (TM) versus TM+Education Material (EM)	192 cancer patients with advanced disease and receiving active treatment	EORTC-QLQ-30 HADS	EORTC-QLQ-30^ǂ^	Once a month over 6 months	Ontological nurses	Yes	Yes	***	****	√√√
					HADS							
					MOS-SS							
					GDS (short form)							
					QARSQ-PH							
					UMPSI							
					GSRE							
					Patient Satisfaction with the Research Program BOMC test;							
Basch et al. (2007) [44]	USA (Memorial Sloan-Kettering Cancer Center, New York)	Prospective pilot study of a patient online self-reporting of toxicity symptoms	107 patients diagnosed with thoracic gynaecologic malignancy starting a new chemotherapy regimen	Feasibility/Pattern of use of a Self-reported online Symptom Track and Reporting (STAR) system Patient satisfaction survey (an exit questionnaire survey) Nursing survey (through an exit survey)	Symptom Track and Reporting (STAR) based on NCI CATAE system	Any clinic visits during 42-wk study period (mean=12, range 1–40) , also possible log in at home during the period	Nurses and study team (n=unreported)	No	Training provide to patients but unreported to staff	**	**	√
Rosenbloom et al. (2007) [34]	USA (Center on Outcomes, Research and Education, Evanston Northwestern Healthcare	RCT: Structured interview and discussion / assessment control / standard care	213 patients with advanced breast, lung or colorectal cancer	Patient HRQoL (Functioning Living Index – Cancer [FLIC]) Patient affect (Brief Profile of Mood States [Brief POMS]) Patient satisfaction [PSQ-III] Clinical treatment changes as reported by nurse (supportive care changes, referrals, ‘other’ clinical changes and changes in standard dose of chemotherapy as a result of PROs)	FACT-G and a single item asking patients whether a particular symptom or problem was better than, worse than, or as expected	Clinic visits at baseline ,and 1, 2,3 and 6 months	Treating nurses (n=not reported)	No	No	***	****	√√√
Weaver et al. (2007) [45]	UK (Oxford Radcliffe Hospitals NHS Trust)	A pilot study of novel mobile phone technology	6 colon cancer patients	Questionnaire on symptoms derived from the Common Terminology Criteria for Adverse Events (CTCAE) grading system	Questionnaire derived from the Common Terminology Criteria for Adverse Events (CTCAE) grading system	Twice daily during the chemotherapy circle (one morning, one evening)	Nurses (n= not reported)	Yes	Yes	**	**	√
Butt et al.(2008) [46]	USA (Center on Outcomes, Research and Education (CORE), Evanton Northwestern Healthcare)	Prospective study to explore the longitudinal screening and management of fatigue, pain, and emotional distress	99 cancer patients with solid tumor of lymphoma undergoing cancer undergoing cancer treatment	FACT-G FACT-Fatigue subscale Brief Pain Inventory (BPI) HADS Structured interview with patients on HRQL and symptom management	FACT-G FACT-Fatigue subscale Brief Pain Inventory (BPI) HADS	Baseline, 1 month and 2 months after the baseline	Doctors and nurses	?	?	**	**	√
Given et al. (2008) [47]	USA( Michigan State University)	RCT: Nurse-Administrated Symptom Management (NASM) vs Automated Telephone Symptom Management (ATSM) intervention	129 breast cancer patients	Outcomes measured at 10–16 wks: 15 symptoms (0–10 scale) Responses & Non-responses of symptoms Time to response	15 symptoms (0–10 scale) Severity of the symptoms	6 contacts or self-reporting (1–4 wk, 6wk, 8wk)	Nurses or ATSM system	Yes	Yes	**	****	√√√
Hilarius et al. (2008) [48]	Netherland (Hospital Pharmacy, Red Cross Hospital, Beverwijk)	A sequential cohort design with repeated measures to evaluate the use of HRQL assessments in daily clinical oncology nursing practice	10 nurses and 219 patients cancer patients with either adjuvant or palliative chemotherapy in a community hospital	Dartmouth Primary Care Cooperative Information Functional Health Assessment (COOPcharts) Patient Management extracted from medical record Patient satisfaction (an exit survey based on PSQ, Form II) Patients’ self-reported HRQL (SF-36, FACT-BCS, FACT-C, FACT-L) Nurse and patient evaluation of the intervention (an exit survey)	EORTC QLQ-C30 EORTC QLQ-BR23 EORTC QLQ-CR38 EORTC QLQ-LC13	Four consecutive visits after baseline for both pre (control arm) and post (intervention arm) with a two-month ‘wash-out’ period	Patients and nurses before consultations	No	Yes	***	***	√√
Mark et al. (2008) [49]	USA (Thomson Healthcare, Washington DC)	A cross-sectional survey of the of both patients’ and health professionals’ experience; A before-after patient chart review	100 cancer patients and 92 health professionals on the experience of The Patient Assessment, Care and Education (PACE) System, including PCM instrument and an education component	Questionnaire survey of 102 providers The patients satisfaction survey (n=100) including 8-item on PCM 200 patient chart reviews (100 charts before and 100 charts after the PACE system)	PCM An education component (not reported in this study)	Each visit to clinic	Clinicians (n=unreported)	No	?	**	***	√√
Kearney et al. (2009) [50]	UK (Cancer Care Research Centre, University of Stirling, Stirling)	RCT: Control group versus intervention group (mobile phone-based remote monitoring of symptoms) over five time points	56 patients with lung, breast or colorectal cancer for each group (total 112 patients)	Paper version of electronic, Mobile phone-based Advanced Symptom Management Systems (ASyMS^©^) based on Common Toxicity Criteria Adverse Events (CTCAE) grading system and the Chemotherapy Symptom Assessment Scale	Mobile phone-based (ASyMS^©^) including chemotherapy-related morbidity of six common symptoms (nausea, vomiting, fatigue, mucositis, hand-foot syndrome and diarrhoea)	Five times including baseline and each of four chemotherapy cycles over a period of 14 days	Doctors only (n=unreported)	Yes	Yes	**	****	√√
Carlson et al. (2010) [51]	Canada (Tom Baker Cancer Centre, University of Calgary, Alberta)	RCT: minimum screening (distress) / full screening / Triage : full screening + referring to appropriate services	585 breast cancer patients + 549 lung cancer patients	Patient distress at 3-month follow-up; Depression and anxiety at 3-month follow-up	Minimum screening: Distress thermometer (DT) Full screening: DT + Psychological scan for cancer part C (PSSCAN)	Baseline	Screening team member (n=unreported)	Yes	Yes	****	****	√√√
Dinkel et al. (2010) [52]	German (Department of Psychotherapy and Psychosomatic Medicine, Technische University Munchen)	Paired comparison : a computerised and a paper version of Stress Index Radio Oncology (SIRO) tool Prospective survey	177 cancer patients in study 1, 273 cancer patients in study 2 (n=142 for computerised version and n=131 for paper version of SIRO) 27 Patients, urses/radiographs and 15 physicians evaluated the screening procedure	Agreement between computer and paper version of SIRO Patient satisfaction; Time need for both modes; Perceived utility; Perceived impact on communication; Perceived impact on patient outcome	SIRO	Any visit	Doctors and nurses (n=unreported)	No	No	***	**	√√
Halkett et al. (2010) [11]	Australian (WA Centre for Cancer and Palliative Care, Curtin University)	Pilot study of using computer touch-screen technology to asses psychological distress in patients	60 patients with various gynaecological cancers	Patient satisfaction with both touch-screen and paper questionnaire; Perceived utility of both modes by patients and health professionals	EORTC QLQ-C30 HADS The Supportive Care Needs Scale The Distress Thermometer Follow questionnaire survey on perceived utility of both modes	Once	Nurses and doctors	Yes	Yes	*	**	√
Ruland et al. (2011) [53]	Norway (Centre for Shared Decision Making and Nursing Research, Oslo University Hospital, Oslo)	RCT: a computer-assisted, interactive tailored assessment (ITPA) with feedback vs ITPA only in oncology practice	145 patients treated for leukaemia or lymphoma	Number of patient symptoms and problems addressed Changes in symptom distress Changes in patients’ need for symptom management support over time, SF-36, Center for Epidemiological Studies Depression Scale (CES-D), Medical Outcome Study Social Support Scale (MOS – SS)	Choice ITPA(19 symptoms (0–4 scale on bothersome) and a severity scale of 0–10)	Every inpatient admission with up to four follow-up visits	Doctors and nurses (n=unreported)	No (as see appropriate)	Yes	***	****	√√√
Velikova et al. (2010) [54]	UK (Cancer Research UK Clinical Centre – Leeds)	RCT: Intervention/control-attention/control in a ratio of 2:1:1	286 cancer patients commencing treatment at the Medical Oncology Clinic at St James Hospital	Medical Care Questionnaire (MCQ): 15-item three subscales: Communication, Coordination, Patient preferences Satisfaction with care Patients’ and physicians’ evaluation of the intervention K-index (Continuity of care: K=(number of visits – number of doctors)/(number of visits −1).	EORTC QOQ-C30 Hospital Anxiety and Depression Scale (HADS)	Regular clinic visit over an average of 6 months	Doctor (n=28)	No	One to one training and manual provided	***	****	√√√
Bainbridge et al.(2011) [55]	Canada (Juravinski Cancer Centre, McMaster University, Hamilton, Ontario)	Survey on the utility of	128 nurses, physicians, and allied health professionals	Perceptions of use and utility of the Edmonton Symptom Assessment System (ESAS) adopted by Ontario’s cancer centres since 2007	ESAS	Every clinic visit	Doctors and nurses	Yes	Yes	*	*	√
Berry et al. (2011) [56]	USA (Dana-Faber Cancer Institute, Boston)	RCT: Intervention / Control	660 cancer patients with various cancer diagnoses and stages at two institutions of a comprehensive cancer centre	1.Audio-recorded content of all communication between clinicians, patients and accompanying friends or family members at each T2 visit (4–6 wks after the treatment)	Patient reported symptoms and quality-of-life (SQLIs) from the Electronic Self-Report Assessment-Cancer (ESRA-C)	Every clinic visit during the study period	Doctors (n=76 principle physicians and other) or incorporated into charts (n=unreported)	No	Yes	***	****	√√√
				2.Clinic visit duration								
				3. Physician exit questionnaire survey								
Cleeland et al. (2011) [20]	USA (MD Anderson Cancer Center, The University of Texas)	RCT: e-mail alert of symptom to patients’ clinical team versus no e-mail alert	79 lung cancer patients receiving thoracotomy	1. Four targeted symptom: pain, distress, disturbed sleep, and shortness of breath, constipation (no fatigue as no effective response) 2. MD Anderson Symptom Inventory (MDASI) at follow-up clinic visit 3. An exit questionnaire survey	Automated telephone calls (IVR system): MDASI (13 common cancer related symptoms)	Twice weekly, up to 4 wks after discharge	Nurses (n=unreported)	Yes	Training to patients provided	**	****	√√
Takeuchi et al. (2011) [57]	UK (St James’s Institute of Oncology, Leeds)	Longitudinal study of data as part of Velikova et al. (2004, 2010) RCT	286 cancer patients commencing treatment at the Medical Oncology Clinic at St James Hospital	Audio-recorded content of Patient-physician communication: Longitudinal impact of PRO intervention; dynamics of communication; association between severity of symptoms/functions and clinic discussion	EORTC QOQ-C30 Hospital Anxiety and Depression Scale (HADS)	Four consecutive visits from baseline	Doctor (n=28)	No	One to one training and manual provided	***	****	√√√

*: Four stars indicate a randomised trial or experimental study; 3 stars indicate a controlled trial, pre–post trial with control (controlled before–after trial), time series, or observational cohort with multivariable adjustment; 2 stars indicate a pre–post trial without control, observational cohort study without multivariable adjustment, cross-sectional study without multivariable adjustment, analysis of time trends without control, or well-designed qualitative study; and 1 star indicates a case series, other qualitative study, or survey (descriptive) study.

√: Three checks indicate great weight in the stratum’s body of evidence, 2 checks indicate moderate weight, and 1 check indicates little weight.

ǂ: EORTC QLQ-C30: European Organisation for Research and Treatment of Cancer Quality of Life (EORTC QLQ) Core Questionnaire; SF-36: The Short Form(36) Health Survey; FACT-G: Functional Assessment of Cancer Therapy-general; EORTC QLQ-BR23: EORTC QLQ Breast Cancer Scale; EORTC QLQ-CR38: EORTC QLQ Colorectal Cancer Scale; EORTC QLQ-LC; EORTC QLQ Lung Cancer Scale; NCI CATAE: National Cancer Institute Common Terminology Criteria for Adverse Events; FACT-BCS: Functional Assessment for Cancer Therapy-Breast Cancer Subscale; FACT-C: Functional Assessment of Cancer Therapy-Colorectal Quality of Life Instrument; FACT-L: Functional Assessment of Cancer Therapy-Lung Cancer Subscale; MOS-SS: Medical Outcomes Study Social Support Survey; GDS: Geriatric Depression Scale (short form); QARSQ-PH: Physical Health subscale of the Older American Resources and Services Questionnaire (OARSQ); UMPSI: Utilisation of Mental Health and Psychosocial Services Instrument; GSRE: Geriatric Schedule of Recent Experience Instrument;

?: No data or unable to classify data available.

### Inclusion and exclusion criteria

The inclusion criteria were: 1) substantial content in presenting empirical evidence on the impact of routinely collected PROs on at least one of the outcomes listed in Table 2; 2) adult cancer patients; 3) conducted in an oncologic setting including inpatient, outpatient and outreach services; and 4) studies using a composite PRO. We defined a composite PRO as those PROs are often based on a well-developed instrument and with an aim for measuring a substantial aspect of patient conditions (or treatment) with at least 4 items. To reflect the demanding and complex nature of evaluating the impact of routine collected PROs, eligible studies included a variety of designs including, but not limited to, randomised controlled trials (RCTs), controlled before-after trials (CBA) and interrupted time series (ITS). ITS designs have a longitudinal character, with repeated measurements and at least three data points before and after the intervention point. Surveys and clinical audits were also included if the studies provided quantitative results relevant to the listed outcomes.

Studies were excluded if they were non-English language articles, opinion and theoretical articles, historical descriptions, review articles, feasibility studies of some PROs collection devices, studies investigating child cancer patients or qualitative studies with no substantial quantitative results on the review endpoints.

### Data extraction and quality assessment

Electronic search results were downloaded into EndNote bibliographic software. Two reviewers independently (JC, LO) screened all titles and abstracts of citations identified by the electronic search, applied the selection criteria to potentially relevant papers, and extracted data from included studies using a standardised form. Any disagreements concerning studies to be included were resolved by consensus.

All studies were classified into two domains. Domain 1 correlated sample characteristics with population wide characteristics, and Domain 2 focused on study design. The data extraction form was adapted from other review studies using the outcome measures discussed above (see Table 4). For each eligible study, a list was made including the leading author, country and jurisdiction, design, sample, outcome measures, the PROs used, times of feedback and intervention, members of medical teams given feedback, management plans offered to teams, and training (see Table 5). All qualifying studies were listed chronologically with the outcome indicators (see Table 3).

**Table 4:** The components, rating criteria and symbol, and categories used in summarising the study evidence in the current study

	**Domain 1**	**Domain 2**	**Global (GRADE)**
Decision Components	Subject of public reporting (or study population) and study participants (sample)	Types of study (i.e. study designs)	Components from Domain 1 & 2 as well as implementation and adherence to intervention, dose–response gradient, precision and validity of the outcomes, uncertainty of direction of the results.
Rating criteria	How well does the study sample represent the study population?	How strong is the study design both in terms of its external and internal validity?	How much weight does the current study add to the evidence-base taking into considerations of all the components above?
Symbol used & categories of rating	1*: no overlap	1*: weakest design	√: little weight
	2*: modest overlap	2*: moderate design	√√: moderate weight
	3*: large overlap	3*: strong design	√√√: great weight
	4*: complete overlap	4*: strongest design	

**Table 5:** The impact and effect sizes of the studies on patients, care providers and organisations*

**Studies**	**Doctor-patient communication**	**Monitor treatment response**	**Detect unrecognised problems**	**Changes to patient health behaviour**	**Changes to patient management**	**Improved patient satisfaction**	**Improved health outcomes**	**Feasibility of the implementation**	**Moderating and subgroup effect**
Trowbridge et al. (1997)[39]			++		++		+ (but no change in PMI)	+++	
Tazenzer et al. (2000)[33]	+++		++		+	-		+++	
McLachlan et al. (2001)[38]	-(no time differences in consultation between two arms)		-(only 37% patients receiving anticancer therapy at baseline)			-	-	+++	+ (on high BDI score subgroup)
Detmar et al. (2002)[37]	+++ (10 out of 12 HRoL measures, especially on social functioning and fatigue)		++		+ (increased patient counselling) +( 25% with family members and primary care physicians)	+ (emotional support)	+ (SF-36)	+++	+ (before-after improvement by intervention group)
						++ (information sharing & communication)			
Mooney et al. (2002)[16]	+++	++	++			+++		++	
Velikova et al. (2004)[36]	+++		++ (64% encounters involving referring to HRoL by physicians)		-(possible due to simple coding between two arms) +(contributed to patient management in 11% of encounters intervention arm).		++(overall quality of life and emotional functioning)	++ (response rate 70%)	+ (more discussion of HRoL subgroup had better outcome within intervention group)
Basch et al. (2005)[41]	+++	+	++			+++	+	++ (65% patient log in before any verbal encouragement)	
Boyes et al. (2006)[35]	+ (50% oncologists in intervention group talked with patients)				-		++ (fewer deliberating symptoms) -(anxiety and depression)	+	-
Hoekstra et al. (2006)[42]	+/−(Only 18% patients used it enhancing communication)			-			++ (lower prevalence in 9 out of 10 symptoms; deteriorated less in 8 out of 10 symptoms)	+	The beneficial effects were pronounced in the deteriorated group.
Korniblith et al. (2006)[43]	+++ (both arms)		++ (more from TM+EM arm)			++ (both arms)	++ (better in TM+EM arm –reduction of psychological distress)	++	
Basch et al. (2007)[44]	+					++		++ (can be improved through reminder)	
Rosenbloom et al. (2007)[34]	-(Possible Ceiling effect)				-	-	-	++	No effect even among the most highly distressed patients
Weaver et al. (2007)[45]	+ (nurse-patient communication)	+	+		+	+	+	++	
Butt et al. (2008)[46]	++	+	+		+	++		++	
Given et al. (2008)[47]		+	+				++ (ATSM more likely to generated responses in symptom management and required less time to do so)	++	+ (Compared with patients receiving combination chemotherapy protocols, those patients treated with single agent had greater response and shorter time to response)
Hilarius et al. (2008)[48]	++	+	++		+	++		++	
Mark et al. (2008)[49]	++	+	+		+	++		++	
Kearney et al. (2009)[50]	++	+	++		++		++ (Fatigue)	+++	
Carlson et al. (2010)[51]							+++ (distress) ++ (decreased depression and anxiety related to referral to services)	+++	
Dinkel et al. (2010)[52]	+				+	+		++	
Halkett et al. (2010)[11]	+ (around 25% of doctors)				+ (10% patients reported changed outcomes)	+ (patients is generally happy with both methods) -(Health professionals found some issues)		+/− (some issues identified but nothing fundamental and patients were generally happy)	
Ruland et al. (2010)[53]			++		++		++	++	
Velikova et al. (2010)[54]	++				(no difference in coordination of care & ‘preferences to see usual doctor’ subscale)	++(86% in intervention vs 29% in the attention-control group)		++	
Bainbridge et al. (2011) [55]	+	+			+			++	+ 89% of nurses and 55% of physicians referred to the ESAS in clinics ‘always’ or ‘ most of the time’
Berry et al. (2011)[56]	++ (25% physician explicitly referred to SQLI summary)							++	++ (the treatment effect on communication is evident on over threshold group on cognitive function, impact on sex and social function)
Cleeland et al. (2011)[20]	++	+	+		+	+	++	++	
Takeuchi et al. (2011)[57]	++ (on symptom but not function)							++	

Note: +++ very strong effect; ++ strong effect; + some effect; +/- uncertain effect; - No effect; blank : untested or reported;

*: Impacts on quality improvement, increased transparency, accountability, public reporting, better population and system performance (monitoring, planning, financing, evaluating, etc) were not listed due to lack of data.

In Domain 1, the routinely collected PROs in particular participants or samples was classified as rated on a 4-point scale representing how closely the participants or samples overlapped with the characteristics and needs of the intended study populations(1 star=very weakly related to 4 stars =very strong related). For example, for a study conducted in the US on a sample of lung cancer patients, the degree of overlap of the study sample with the characteristics of lung cancer patients in the US overall was assessed by considering the study setting, sample size and sampling frame, response rate, loss-to-follow-up, and characteristics of the study sample. In Domain 2, study design was classified and rated on 4 categories with 1 star indicating the weakest design and 4 stars indicating the strongest design. Four stars indicated a randomised trial or experimental study; 3 stars indicated a controlled trial, pre–post trial with control (controlled before–after trial), time series, or observational cohort with multivariable adjustment; 2 stars indicated a pre–post trial without control, observational cohort study without multivariable adjustment, cross-sectional study without multivariable adjustment, analysis of time trends without control, or well-designed qualitative study; and 1 star indicated a case series, other qualitative study, or survey (descriptive) study.

Revised appraisal criteria were adapted from the guidelines on the assessment of quality improvement interventions [58,59]. A global rating was also created using the Grading of Recommendations, Assessment, Development, and Evaluation (GRADE) system [60]. The British Medical Journal has recommended the GRADE system since 2006 for grading evidence when submitting a clinical guidelines article. It has multiple advantages and is useful for systematic reviews and health technology assessments, as well as for evaluating research on clinical guidelines. The global rating created in the current study was based on the integration of the Domain 1 and Domain 2 ratings, as well as the intervention fidelity (the degree of success of the interventional strategy, the patients’ and providers’ adherence to the intervention strategy), dose–response gradient, precision and validity of outcomes (potential confounding factors and biases), and uncertainty of the direction of results. The global rating was divided into three categories; indication that the study should carry great (3 checks), moderate (2 checks), or little (1 check) weight when considering the strength of evidence (see Table 4). Any experimental research that is reported in the manuscript was performed with the approval of an appropriate ethics committee.

No attempt was made to quantitatively synthesize the results as the data were too heterogeneous to support pooling.

## Results

**Figure 3:**
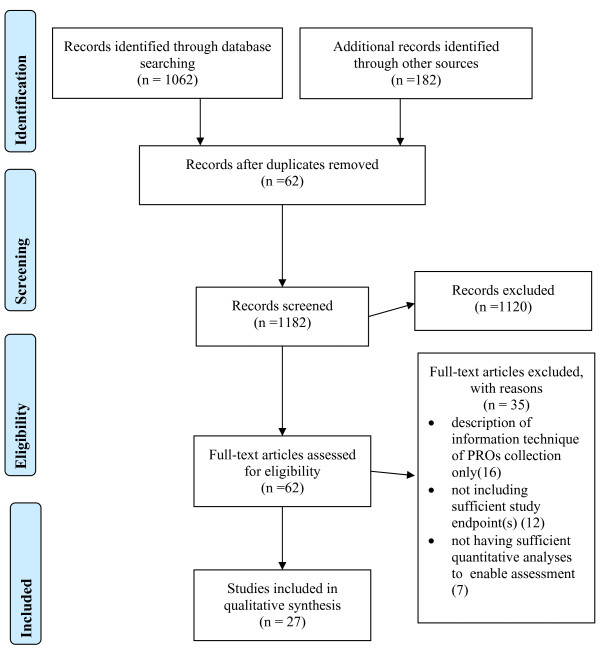
PRISMA Flow Diagram illustrating the systematic review process from electronic searching through to study inclusion.

The multi-method search strategy yielded 27 publications that were eligible to be included in the review – a significant increase compared with most of the recent reviews. The detailed flowchart of the search strategies and its relative results was presented (Figure 3). The results and conclusions drawn were based on the 27 studies included in the analyses despite the large number of full-text articles extracted.

Of the 27 publications, 16 were identified as randomised controlled trials, 2 as before-after studies and 9 observational studies with 11 studies published before 2009. The characteristics and quality of the studies are presented in Table 5 with their impact on outcome indicators presented in Table 3. As Trowbridge et al. (1997) was the only article in the 1990s included in two of the previous reviews, it was listed in the summation tables for the purpose of comparison.

### Overview of study quality

There has been a marked increase in the volume and quality of the studies published recently in this area. Of the 16 randomised controlled trials included in this review, 7 were published between 2010 and 2011. The quality of studies published since 2010 is also demonstrably improved with much larger sample sizes, including 3 trials [54,57,61] with a sample size greater than 200 and 2 trials with a sample size over 580 [51,56].

However, despite the increased volume and improved quality of the studies, there remains a lack of large cluster randomised controlled design studies, as recommended by Fayers [62] who argues that cluster RCTs are well suited to overcome the limitations of simple RCTs. It is well-known that system intervention trials such as routine collection of PROs, and feedback to the clinicians and systems, are prone to cross-contamination and to introducing investigator and participant biases. Two recently published studies [54,57] were the continuation of an earlier study published by Velikova et al. (2004) [36]. Most studies reviewed did not systematically examine outcomes and mechanisms, and placed more emphasis on processes rather than outcome measures [25]. All studies were conducted in a limited setting (often in a single centre) thus restricting the generalisation of the findings.

No studies have adopted a comprehensive theoretical model and framework, despite the repeated demand from leading researchers in the area [25,63-65]. All studies focused on the patient and health professional level within the clinic setting. No study to date has examined the impact of collecting PROs on health care organisations, health system improvement, quality improvement or population health at a system or societal level.

### Overview of study findings

#### Impact on patient-provider communication

Across the 27 studies included in this review, 4 studies [39,47,51,53] did not examine or report the effect of a routinely collected PRO on patient-provider communication. Among the 23 studies that did report such an impact, 21 studies (91.3%) reported a positive effect which included well-designed and conducted large RCTs [33,36,37,54,56,57]. One study reported no significant improvement of patient-provider communication possibly due to a lower severity level of cancer patients (only 37% of patients received anticancer therapy, hence the reduced need for communication for the treatment) [38]. Another study reporting a negative effect had an already high communication level at baseline (hence a ceiling effect leaving little room for further improvement) [34].

#### Impact on monitoring treatment response

Despite most of included 27 studies did not explicitly state their study objectives as to examine the impact on monitoring treatment response, 11 of the 27 studies did report an outcome (Table 3) [16,20,36,41,45-51]. All 11 studies found a strong or modest effect of implementing PROs on the increased monitoring activities of treatment response. The strongest effect occurred in the studies that focused on the monitoring of patient symptoms, side effects and toxicity during and after chemotherapy for the outpatients. In particular, the real-time, patient reported symptoms and toxicity (through innovative mobile phone-based, web-based or IVR systems) significantly improved the monitoring of treatment response.

#### Impact on detecting unrecognised problems

Although the idea of routinely collected PROs may provide better opportunities for services providers (as well as patients) to detect unrecognised problems through growing awareness, improved communication and monitoring seems intuitively plausible, only 16 out of 27 studies reported some results related to the detection of unrecognised problems (Table 3). Amongst the 16 studies, 15 studies [16,20,33,36,37,39,41,43],[45-50,53] reported either a strong or moderate positive impact on detecting unrecognised problems. However, a study by McLachlan and colleagues [38] did not find any difference between the intervention arm and control arm.

#### Impact on changes to patient health behaviour

No study to date has provided a systematic evaluation on the impact of collecting PROs on changes to patient health behaviour. It is unknown whether and how patient health behaviours have been changed.

#### Impact on changes to patient management

Amongst 17 studies that provided some results of changes to patient management, 13 studies [11,20,33,37,39,45,46,48-50],[52,53,55] reported either a strong or modest positive effect on the changes to patient management whilst 4 studies [34-36,54] found no such effect. However, it is worth noting that 10 studies did not provide any information about the changes to patient management and there were often less complete descriptions of the results on patient management when reported.

#### Impact on patient satisfaction

Among the 16 studies that reported results related to the impact on patient satisfaction, 13 studies [11,16,20,37,41,43-46,48,49],[52,54] reported a very strong to moderate positive effect on improved patient satisfaction. For the three studies [33,34,38] that did not find such a positive effect, one study [33] reported a possible ceiling effect meaning that both the intervention group and control group had a very high baseline patient satisfaction level potentially impeding any demonstration of a significant difference between two arms during the follow-up period.

#### Impact on health outcomes

Amongst the 15 studies that reported some results related to the impact on health outcomes, 13 studies [20,35-37,39,41-43,45,47,50,51],[53] reported some positive improvement, ranging from modest to strong, while two studies [34,38] failed to find any such effect. It appears that symptoms, side effects and toxicity are most likely to be improved, followed by emotional wellbeing. There is little evidence on the improvement of both overall HRQOLs as well as social wellbeing.

#### Impact on quality improvement, transparency, accountability and public reporting, and on better system performances (monitoring, planning, financing, evaluating, responding)

No study to date has provided a meaningful, explicit framework nor relevant evidence on these endpoints.

#### Overall strength and direction of evidence

Overall, there is strong evidence supporting the notion that routinely collected PROs, with feedback, improves patient-provider communication and increases patient satisfaction (Table 6). There is some evidence to support the notion that it improves the monitoring of treatment responses and detection of unrecognised problems, and there is weak but positive evidence that, over time, it leads to changes in patient management. Despite some encouraging results, there is still a great degree of uncertainty regarding the impact of routinely collected PROs, with feedback, on patient health outcomes. There is little or no evidence that it has led to significant positive improvements in quality improvement, transparency, accountability, and public reporting, or in system performance at a population health or societal level. Apart from clinical trials and clinical practice, its impact on health services research and population health is largely unknown.

**Table 6:** The overall strength and direction of evidence

**Results**	**Strength and direction of evidence**
Patient-provider communication	+++
Monitor treatment response	++
Detect unrecognised problems	++
Changes to patient health behaviour	n/a
Changes to patient management	+
Improved patient satisfaction	+++
Improved health outcomes	+/0
Strong & effective quality improvement	n/a
Increased transparency, accountability, public reporting	n/a
Better system performance (monitoring, planning, financing, evaluating, responding)	n/a

Note: ++++: the strongest positive effect; xxxx: the strongest negative effect; n/a: not available; 0: mutual (no significant effect).

#### Potential moderating factors and links between routine PRO collection (with feedback) and patient outcomes

Although the evidence is limited, it appears that routine collected PROs with sufficient intensity of feedback (multiple times over a sustained period of time) [13,39,44,54], targeting multiple stakeholders (doctors, nurses, allied health workers, as well as patients) [35,52] with simple, clear, graphical and longitudinal meaningful interpretation of the results, and providing sufficient training for both health professionals and patients [20,57], are critical links between an intervention and the intended outcomes. There is also evidence to suggest that for some complex issues such as depression and low social functioning, routine screening and feedback may need to be integrated with other strategies such as decision-making aids, education, clear management plans and clinical pathways including referrals, in order to change patient outcomes [43,49,51]. There is preliminary evidence that some of the impacts of PROs may be more pronounced amongst subgroups with more severe problems at baseline (e.g. depression, symptoms) [38,42,65]. More studies are needed to fully understanding these mediating and moderating effects.

## Discussion

There is very strong evidence in supporting the notion that routine collected PROs with timely feedback enhances patient-provider communication. This current study finding of a positive effect on patient-provider communication is consistent with previous reviews conducted in both cancer [26] and non-cancer settings [25,27,28]. There is also strong evidence to support the notation that routine collected PROs significantly improved the monitoring of treatment response.

There is reasonably strong evidence in supporting the notation that routine collected PROs are helpful in identifying unrecognised problems in a large variety of settings. Within studies that reported, to some extent, the results related to unrecognised problems, there is a need for the development of more comprehensive and valid measures. Such a change in the measures would contribute towards understanding specifically the PROs’ impact on identifying the underreported and unrecognised problems for different cancer patients at different settings.

Overall, there is reasonable evidence in favouring the hypothesis that implementing a routine collected PROs system brings positive changes to patient management in the settings where a patient management plan is integrated with a routine collection of PROs. It appears that the simple routine feedback of PROs may not be sufficient to improve patient management and outcomes [48]. Other necessary resources may be needed such as education, referral services and a detailed patient management plan following the PROs [43]. There is also a need to develop better measures of change to patient management as it is often complex and difficult to quantify [57].

There is strong evidence to support the notation that routine collected PROs with timely feedback significantly enhance patient’s experience and satisfaction. There may be other improved experience and satisfaction in other stakeholders such as patients’ family members, caregivers, as well as health professionals that were not measured or unreported. Future research into furthering the understanding of stakeholder experience after implementing routine collected PROs would be desirable.

Although positive evidence in supporting the notion that routine collected PROs may improve health outcomes is weak, this finding needs to be confirmed by better designed studies covering a large set of well-developed outcome measures. There is also a need to understand the impact on long-term health outcomes such as survival rate. Most of the studies included in this review did not focus on health outcomes and some of the positive improvement on the outcomes only occurred on selective measures. It is not clear how these positive improvements can be generalised across different settings.

There is a variety of models on how to routinely collect PROs and how to feed back the data to different stakeholders. Given that cancer patients are vastly different given their background, type and stage of cancer, prognosis, treatment, and the positions at the life course continuum, precaution should be exercised when attempting to apply the general observation above to each and every different setting. For example, recent studies demonstrated a positive impact of routine collected PROs on symptom control through either web-based or mobile phone based approach. However, such positive impacts were less pronounced on HRQOL.

### Limitations of the current review

Our review has several limitations. First, there was no attempt made to contact the authors to ask for potential unpublished data on the topic. Thus, there may be chance of missing some grey literature or the studies that under preparation for publication. Second, given the multitude of endpoints included, and different types of studies involved, the assessment of eligibility for inclusion of potential studies required some degree of subjective judgement. Third, our application of GRADE system was rather simplistic restricted by large number of endpoints and variability of studies included. These limitations may give rise to some uncertainty in terms of synthesis of the results. Fourth, our study follows a systematic review approach with inclusion of both experimental trials and quantitative observational studies. However, we did not include qualitative studies in our review which may provide additional insight into the questions raised. This is particularly relevant with respect to questions 2 and 3 as there was very little quantitative evidence from the included studies. It is important to note that despite efforts to formulate the review endpoints based on solid and well-established causal and theoretical frameworks (providing insight into not only if but also how the introduction of PROs affects patient outcomes), the causal mechanisms and process endpoints included in the current review are by no means exhaustive. There may be other important causal mechanisms that could be benefited from a realist review approach [66].

## Conclusions

There is growing evidence supporting the routine collection of PRO to enable better and patient-centred care, especially in cancer settings. Despite the strong evidence in supporting the notion that the well-implemented routine collection of PROs enhances patient-provider communication and improves patient satisfaction, and growing evidence supporting ideas that it also improves the monitoring of treatment response and the detection of the unrecognised problems, the evidence-base was weak for its impact on changes to patient management and improved health outcomes and non-existent for changes to patient health behaviour, strong and effective quality improvement, increased transparency, accountability, public reporting and better health care system performance. These evidence gaps require further committed and well-planned research in addition to the well-accepted PROs. Decision-making agencies have been well positioned for leverage on the rapid advancement of different PRO models, the application of the item response theory and computer adapted test in developing PROs, and on the acceptance of such technology by patients and health professionals over the last decade. The real-time and routinely collected PROs will enable the development of a rapid learning health system with the potential to advance our knowledge of drug development, unsurpassed models of cancer patient care and a more patient-centred health care system.

## Competing interests

The authors declare that they have no competing interests.

## Authors’ contributions

JC contributed to the conceptualisation and design of the study. JC, LO, SH conducted the review. JC prepared the first draft of the paper and all other authors contributed to and approved the final manuscript.

## Pre-publication history

The pre-publication history for this paper can be accessed here:

http://www.biomedcentral.com/1472-6963/13/211/prepub

## Supplementary Material

Additional file 1 **Appendix 1.** Full text search strategies used in Scopus. Click here for file
