# Supplementary material for: A systematic review of the impact of routine collection of patient reported outcome measures on patients, providers and health organisations in an oncologic setting
Source: BMC Health Serv Res. 2013 Jun 11;13:211. doi: 10.1186/1472-6963-13-211 (PMC3700832; doi:10.1186/1472-6963-13-211)
Supplement: Additional file 1 — Appendix 1. Full text search strategies used in Scopus. [file 1472-6963-13-211-S1.doc]

## Appendix 1: Full text search strategies used in Scopus

Strategy A: A more extensive search covering a larger literature

(TITLE-ABS-KEY (patient reported outcome) OR TITLE-ABS-KEY (self-reported) OR TITLE-ABS-KEY (self-assessed)OR TITLE(routine) )
AND (TITLE-ABS-KEY(quality of life) OR TITLE-ABS-KEY(symptom) OR TITLE-ABS-KEY(functional status) OR TITLE-ABS-KEY(health status) OR TITLE-ABS-KEY(patient satisfaction) OR TITLE-ABS-KEY(unmet need*))
AND (KEY(neoplasm) OR KEY(cancer))
AND PUBYEAR > 1999

Strategy B: A more restricted search with some search terms being restricted to titles

(TITLE(patient reported outcome) OR TITLE(self-reported) OR TITLE(self-assessed)OR TITLE(routine) )
AND (TITLE-ABS-KEY(quality of life) OR TITLE-ABS-KEY(symptom) OR TITLE-ABS-KEY(functional status) OR TITLE-ABS-KEY(health status) OR TITLE-ABS-KEY(patient satisfaction) OR TITLE-ABS-KEY(unmet need*))
AND (KEY(neoplasm) OR KEY(cancer))
AND PUBYEAR > 1999
